# Sturge-weber syndrome: a case report with persistent headache

**DOI:** 10.11604/pamj.2014.18.87.3346

**Published:** 2014-05-26

**Authors:** Ece Balkuv, Nihal Isik, Ilknur Aydin Canturk, Nejat Isik, Recep Basaran

**Affiliations:** 1 Istanbul Medeniyet University Goztepe Education and Research Hospital, Neurology Department, Istanbul, Turkey; 2 Istanbul Medeniyet University Goztepe Training and Research Hospital, Neurosurgery Department, Istanbul, Turkey; 3 Lutfi Kirdar Kartal Training and Research Hospital, Neurosurgery Department, Istanbul, Turkey

**Keywords:** Sturge-weber syndrome, congenital disorder, vascular nevus, angioma

## Abstract

Sturge-Weber syndrome (SWS) is a rare congenital disorder characterized by a facial vascular nevus associated with an ipsilateral leptomeningeal angioma. Headache is a rare component of SWS and when it occurs it usually occurs as a migraine-like headache. We aimed to present a SWS patient with episodic tension type headache and to draw attention in different types of headaches that can be seen in SWS. A 21 year old female patient with the diagnosis of SWS was suffering from severe headaches. At her physical examination a facial nevus -occurred due to choroid angioma- was observed. On her neurological examination a mild asymmetry of upper extremities was visible. She had a 2 year history of frequent non-pulsating headaches. There was no nausea or aura like symptoms accompanying the headache. Headaches were lasting for hours. The pain was bilateral and pressing in quality. SWS are a very rare and challenging disease for both the patients and their families. Usually migraine type headache is seen in SWS but it should not be forgotten that more generalized headaches like tension type may also be seen.

## Introduction

Sturge-Weber syndrome (SWS) is a rare congenital disorder characterized by a facial vascular nevus associated with an ipsilateral leptomeningeal angioma most often involving the occipital and posterior parietal lobes. Seizures are the usual neurological manifestation in up to%80 of patients [1]. Other manifestations related to this syndrome are hemiparesis, glaucoma and mental retardation [2]. Neurological involvement is related to the presence of leptomeningeal angiomas involving the pia mater. It is believed that this syndrome's possible cause is the formation of a vascular plexus around the cephalic portion of the neural tube [3]. Estimated incidence of SWS is 1 per 50.000 live births (Thomas-sohl, vaslow and maria 2004) [4].

Headache is a rare component of SWS and when it occurs it usually occurs as a migraine-like headache [5]. All cases of headaches accompanying SWS were migraine-like headaches in the literature. In this case we aimed to present a SWS patient with episodic tension type headache (IHS criteria section 2.2). Our purpose was to draw attention in different types of headaches that can be seen in SWS.

## Patient and observation

A 21 year old female patient with the diagnosis of SWS suffering from headaches admitted to our clinic. She had a 2 year history of frequent non-pulsating headaches. Her headache was relieving with non-steroidal anti-inflammatory drugs and was not worsening with physical activity. There was no nausea or aura like symptoms accompanying the headache. Headaches were lasting for hours. The pain was bilateral, generalized and pressing in quality. The family history for headache was negative.

She had a history of seizures occurring in the fifteenth day of life described as attacks of tonic clonic contractions and that's when she was diagnosed with SWS. At the age of 6 she had a history of callosotomy to control her seizures. At the age of 18 during a laser treatment done in order to get rid of her port wine birthmark she had her first seizure since callosotomy. After that she was prescribed carbamazepine 400 mg at daily dose and never had a seizure since then. According to the story taken from her parents even though she had a normal development at infancy she barely graduated from elementary school and she's hardly literate.

There was nothing significant on her family history except for her elder sister's port wine stain on her face. The elder sister had no feature of SWS and no researches were done regarding her stain. She was inscribed daily doses of ketiapin 25 mg for anxiety disorder and venlafaxine 75 mg for both anxiety disorder and the chronic headaches. She was also inscribed NSAID drugs. After the first week of this treatment her headaches were slightly decreased by heaviness but the frequency was the same.

**Figure 1:**
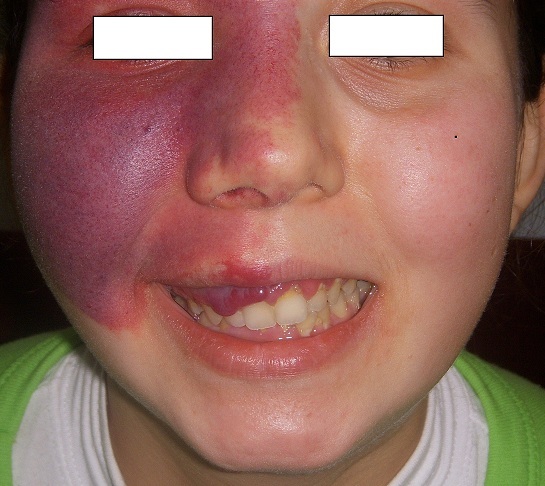
Congestive skin lesion (port wine stain) is seen on right nasal, periorbital, frontal and maxillary areas corresponding to trigeminal nevre area. Also evident gingival hyperplasia is remarkable at the same side

At her physical examination a facial nevus -occurred due to choroid angioma- on the right forehead, right eyelid, nasal wing and the cheek was observed (Figure 1). Intra oral examination showed a right sided overgrowth of gingiva. Gingival overgrowth was bright red in color and showed blanching on applying pressure suggesting angiomatous enlargement (Figure 1). On her extremities a mild asymmetry was visible. Her left arm and leg was slightly smaller in portions and showed hemiparesis both in the upper and lower extremities of the same size. On her ophthalmological evaluation she was diagnosed with glaucoma of the right eye. On her psychiatric examination she showed signs of anxiety disorder. Her neurological examination was not remarkable except for her hemiparesis.

**Figure 2:**
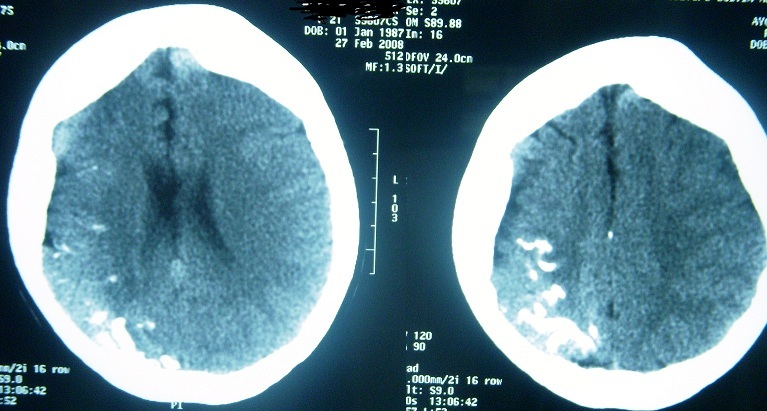
Axial cranial BT image shows corticosubcortical calcifications at right parietal area and calvarium is seen thickened at frontal and right temporal areas

**Figure 3:**
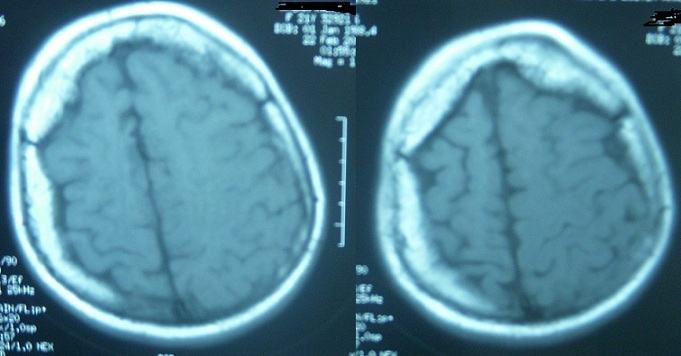
Axial T1 weighted cranial MRI shows right calvarial thickness compared to the left and right hemisphere is asymmetrically smaller then the left

**Figure 4:**
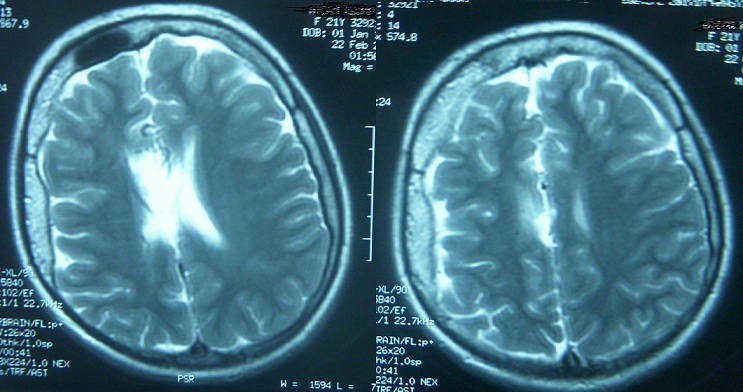
T2 weighted MRI shows extensive venous formations around corpus of right lateral ventricle and at Gallen vein localization and widespread vascular formations are seen at perivascular space, anterior to third ventricle at Willis polygon localization and at right temporooccipital area at quadrigeminal cistern localization

Cranial CT scans showed diffuse atrophy in the right hemisphere and irregular double-contoured gyriform cortical calcifications in the right occipital area (Figure 2). Gadolinium enhanced brain MRI revealed multiple dilated pial venous vascular structures on right hemisphere also with the diffuse atrophy on the same side. Axial T1 weighted cranial MRI shows right calvarial thickness compared to the left and right hemisphere is asymmetrically smaller than the left (Figure 3). In addition to that, T2 weighted MRI shows extensive venous formations around corpus of right lateral ventricle and at Gallen vein localization and widespread vascular formations are seen at perivascular space, anterior to third ventricle at Willis polygon localization and at right temporooccipital area at quadrigeminal cistern localization (Figure 4). She was performed a proteus intelligence test in which she had 75 points and accepted as mildly mentally retarded. Proteus intelligence test in which she had 75 points and accepted as mildly mentally retarded.

## Discussion

Sturge Weber Syndrome manifests as three distinct types, distinguished by the presence or absence of facial and/or central nervous system angioma and glaucoma [4]. Type 1 includes facial capillary malformation (port-wine birthmark), vascular malformation of the brain (intracranial leptomeningeal angioma), and possible glaucoma. Type 2 is believed to be the most common presentation and involves facial angioma and the possibility of glaucoma, but an absence of brain involvement. Type 3 is characterized by the presence of leptomeningeal angioma without port-wine birthmark, and typically without the development of glaucoma or other eye abnormalities [4]. Our case is type 1 SWS.

The prevalence of migraine in SWS was seen to be higher than in normal population [6]. Conversely, no case of headache was reported in a long term outcome study regarding 52 adults with SWS [7]. The headache secondary to encephalo trigeminal angiomatosis was not included in the first edition of the International Headache Society classification. In the second edition this headache was classified in the secondary headaches attributed to cranial or cervical vascular disorders [8].

In our case migraine-like symptoms were absent. At another study conducted at the Neurology and Headache Centre with 71 subjects the prevalence of recurrent headache was% 44, including migraine (%28), headache related to glaucoma (%8), chronic tension-type headache (%4), episodic tension type headache (%1) and unclassifiable headache (%3) [6]. The sex ratio was approximately equal. Headache meeting IHS criteria for migraine was seen in 28% (equally among males and females) compared to 17% in females and 5% in males in the general population [6]. Another type of headache seen often in SWS patients is postictal headaches [6]. Our subject was fulfilling the frequent episodic tension type headache criteria of the IHS (section 2.2).

Various imaging findings such as gyriform calcifications, leptomeningeal enhancement atrophy of the ipsilateral hemisphere, thickened calvarium, ipsilateral choroid plexus enlargement, enlargement of paranasal sinuses and mastoid air cells and enlargement of deep veins are seen in SWS. The most specific finding is leptomeningeal enhancement. Gyriform calcification, atrophy of the ipsilateral hemisphere, and ipsilateral choroid plexus enlargement are also seen very common [9]. In our case almost all of the specific features of the disease are seen including ipsilateral curvilinear calcifications, hemi atrophy, ipsilateral choroid plexus enlargement, ipsilaterally thickened calvarium and ipsilateral multiple dilated venous structures. Usually all radiological changes happen at the same side with the cutaneous angioma except for one case published at the Journal of Child Neurology at 2003 [10]. Another important subject that has to be mentioned on the prompting of this case is the importance of callosotomy at the proper age. This “proper age” is not defined in any literature but there are some important hypothesis about it. Steinbok and colleagues analyzed 116 subjects from eight centers. Seizure onset was in the first year of life in 82%, and mean age at first surgery was 15.8 months (1-35 months). Results showed that epilepsy surgery in children younger than 3 years of age is relatively safe and is effective in controlling seizure and early surgery impact development positively [11]. In another study examining 20 patients, none of the patients showed any aggravation of cognitive impairment following surgery and none of those who were operated on early presented with severe mental retardation [12]. Our subject was operated at age 6 and it is reasonable may to wonder could she have higher scores on her IQ test if she was operated at an earlier age.

## Conclusion

SWS are a very rare and challenging disease for both the patients and their families. This illness effects many systems thus have many features. Usually migraine type headache is seen in SWS but it should not be forgotten that tension type headaches may also be seen. Treatment of this rare type of headache is very challenging
